# The Therapeutic Efficacy of Drugs Targeting the NO‐sGC‐cGMP Pathway in Treatment of Patients With Chronic Thromboembolic Pulmonary Hypertension: A Systematic Review

**DOI:** 10.1002/pul2.70337

**Published:** 2026-07-15

**Authors:** Abdullah A. Alqarni, Rawan H. Aljedani, Abdulelah M. Aldhahir, Ahmed H. Alasimi, Abdulkareem A. AlGarni, Jaber S. Alqahtani, Saeed Mardy Alghamdi, Rayan A. Siraj, Aminah Mengash, Hassan Alwafi

**Affiliations:** ^1^ Department of Respiratory Therapy, Faculty of Medical Rehabilitation Sciences King Abdulaziz University Jeddah Saudi Arabia; ^2^ Respiratory Therapy Unit King Abdulaziz University Hospital Jeddah Saudi Arabia; ^3^ Department of Respiratory Therapy Armed Forces Hospitals Taif Region Taif Saudi Arabia; ^4^ Respiratory Therapy Program, Department of Nursing, College of Nursing and Health Sciences Jazan University Jazan Saudi Arabia; ^5^ National Heart and Lung Institute Imperial College London London UK; ^6^ College of Applied Medical Sciences King Saud bin Abdulaziz University for Health Sciences Al Ahsa Saudi Arabia; ^7^ King Abdulaziz Hospital, The Ministry of National Guard Health Affairs Al Ahsa Saudi Arabia; ^8^ King Abdullah International Medical Research Center (KAIMRC), Eastern Region Al Ahsa Saudi Arabia; ^9^ Department of Respiratory Care Prince Sultan Military College of Health Sciences Dammam Saudi Arabia; ^10^ Clinical Technology Department, Respiratory Care Program, Faculty of Applied Medical Sciences Umm Al‐Qura University Makkah Saudi Arabia; ^11^ Department of Respiratory Therapy, College of Applied Medical Sciences King Faisal University Al‐Ahsa Saudi Arabia; ^12^ Department of Clinical Pharmacology and Toxicology, Faculty of Medicine Umm Al‐Qura University Makkah Saudi Arabia

**Keywords:** COPD, group 3 PH, nitric oxide pathway, PDE5 inhibitors, phosphodiesterase‐5 (PDE5) inhibitors, pulmonary hypertension, sGC stimulators, sildenafil, soluble guanylate cyclase stimulators, tadalafil

## Abstract

Chronic thromboembolic pulmonary hypertension (CTEPH) is a distinct and potentially curable form of pulmonary hypertension; however, a substantial proportion of patients remain inoperable or experience persistent or recurrent disease. Pharmacological therapies targeting the nitric oxide–soluble guanylate cyclase–cyclic guanosine monophosphate (NO–sGC–cGMP) pathway have emerged as promising treatment options. Therefore, this systematic review aimed to comprehensively evaluate the therapeutic efficacy of drugs targeting the NO–sGC–cGMP pathway on clinical outcomes in patients with CTEPH. We conducted a comprehensive search of electronic databases, including Embase, MEDLINE, Cochrane Library, and Scopus databases from inception to December 1, 2025. Studies involving adult patients with confirmed CTEPH receiving NO–sGC–cGMP pathway‐targeted therapies were included. Exclusion criteria encompassed case reports, systematic reviews, review articles, conference abstracts with no full text (since they were not peer‐reviewed), non‐full‐text articles, non‐English manuscripts, opinion articles, and book chapters. Risk of bias was assessed using the Cochrane RoB 2 tool and the Newcastle–Ottawa Scale. A total of 23 studies involving 2815 patients were included. The most frequently investigated agents were riociguat and sildenafil. Fourteen of 15 studies demonstrated significant improvements in exercise capacity, with consistent findings reported in major trials such as CHEST‐1 and its long‐term extension, CHEST‐2. All 16 studies assessing pulmonary hemodynamics reported significant improvements in mPAP, PVR, and CO. Additionally, 12 studies showed reductions in NT‐proBNP levels and improvements in WHO‐FC, indicating enhanced functional status. Quality of life was assessed in two studies, both of which demonstrated favorable outcomes. Furthermore, three studies reported improvements in right ventricular function and risk stratification. Pharmacological agents targeting the NO–sGC–cGMP pathway, particularly riociguat and sildenafil, demonstrate consistent improvements in exercise capacity, pulmonary hemodynamics, functional status, and biomarkers in patients with CTEPH. These findings support their role as effective therapeutic options, especially in inoperable or persistent CTEPH. Nevertheless, further large‐scale, high‐quality randomized controlled trials are warranted to strengthen the evidence base and optimize treatment strategies.

Systematic review registration: CRD42024510274, https://www.crd.york.ac.uk/PROSPERO/view/CRD42024510274.

**Abbreviations**
6MWD: 6‐min walk distance
BPA: balloon pulmonary angioplasty
cGMP: cyclic guanosine monophosphate
CO: cardiac output
CTEPH: chronic thromboembolic pulmonary hypertension
mPAP: mean pulmonary arterial pressure
NO: nitric oxide
NT‐proBNP: N‐terminal pro‐brain natriuretic peptide
PAC: pulmonary arterial compliance
PDE5: phosphodiesterase type 5
PEA: pulmonary endarterectomy
PVR: pulmonary vascular resistance
RCTs: randomized controlled trials
sGC: soluble guanylate cyclase
WHO‐FC: World Health Organization functional class

## 1 Introduction

Pulmonary hypertension (PH) is a progressive cardiopulmonary disorder characterized by elevated pulmonary arterial pressure and increased pulmonary vascular resistance, which can culminate in right ventricular failure and premature mortality [1]. Contemporary classification frameworks group PH into five major categories based on underlying etiology, pathobiology, and treatment approach [2]. Chronic thromboembolic pulmonary hypertension (CTEPH), classified as group 4 PH, results from persistent obstruction of the pulmonary vasculature by organized thromboembolic material, accompanied by secondary microvascular remodeling and small‐vessel arteriopathy [3].

CTEPH is a potentially curable form of PH. However, a substantial proportion of patients are not eligible for surgery or develop persistent or recurrent PH after pulmonary endarterectomy (PEA) [4, 5]. These patients frequently experience marked functional limitation, reduced exercise capacity, impaired quality of life, and excess mortality [6]. Balloon pulmonary angioplasty (BPA) has expanded interventional options for selected patients, yet pharmacological therapy remains central for inoperable disease and for residual PH following intervention [7].

The nitric oxide–soluble guanylate cyclase–cyclic guanosine monophosphate (NO–sGC–cGMP) signaling pathway is a key regulator of pulmonary vascular tone, endothelial function, and vascular remodeling [8]. Endogenous nitric oxide activates sGC, increasing intracellular cGMP and promoting vasodilation while also exerting antiproliferative and antithrombotic effects on the pulmonary vasculature [9]. Dysfunction of this pathway has been described across multiple forms of PH, including CTEPH, supporting a mechanistic rationale for therapeutic targeting [10].

Therapies that enhance NO–sGC–cGMP signaling include phosphodiesterase type 5 (PDE5) inhibitors, which reduce cGMP degradation, and sGC stimulators, which increase cGMP generation and may retain activity even when nitric oxide bioavailability is reduced [11]. In CTEPH, riociguat is the only pharmacotherapy with regulatory approval specifically for inoperable disease and for persistent/recurrent PH after PEA, supported by randomized trial evidence demonstrating improvements in pulmonary hemodynamics and exercise capacity [12]. Nevertheless, variability in treatment response, heterogeneity in clinical phenotype, and evolving use of combination or sequential strategies continue to motivate careful synthesis of the overall evidence base [13].

In parallel, additional approaches to augment the NO–sGC–cGMP axis, including investigational sGC modulators and inhaled nitric oxide–based strategies, have been explored in PH populations and may hold relevance for selected CTEPH contexts, raising questions about optimal patient selection, timing, and outcomes [14, 15]. Despite expanding clinical use of pathway‐targeted therapy, the magnitude of benefit, comparative effectiveness across drug classes, and impacts on hemodynamics, gas exchange, and functional outcomes in CTEPH remain incompletely defined. Therefore, this systematic review aimed to evaluate the therapeutic efficacy of drugs targeting the NO–sGC–cGMP pathway in patients with CTEPH, with emphasis on exercise capacity, pulmonary hemodynamics, World Health Organization functional class (WHO‐FC), N‐terminal pro‐brain natriuretic peptide (NT‐proBNP) levels, quality of life, and right ventricular function.

## 2 Materials and Methods

The protocol for this systematic review was prospectively registered on PROSPERO (registration number: CRD42024510274). The review was conducted in accordance with the Preferred Reporting Items for Systematic Reviews and Meta‐Analyses (PRISMA) guidelines for the systematic review [16].

The search strategy was developed using the PICO framework to formulate focused clinical questions: P (population): adults with a confirmed diagnosis of CTEPH; I (intervention): drugs targeting the NO–sGC–cGMP pathway; C (comparison): placebo or usual care; and O (outcomes): exercise capacity, pulmonary hemodynamics, WHO‐FC, NT‐proBNP levels, quality of life, and right ventricular function.

### 2.1 Eligibility Criteria

Studies were selected according to predefined inclusion and exclusion criteria:

#### 2.1.1 Inclusion Criteria

- Adults aged > 18 years with a confirmed diagnosis of CTEPH
- Studies that reported drugs targeting the NO–sGC–cGMP pathway (e.g., PDE5 inhibitor, sGC stimulator, and inhaled nitric oxide) as comparators.
- Study designs include randomized controlled trials, prospective and retrospective observational studies, phase II–III multicentre trials, mechanistic studies, open‐label extension and uncontrolled trials, and post hoc analyses of the PATENT and CHEST studies.
- No restriction on CTEPH severity.

#### 2.1.2 Exclusion Criteria

- Studies that did not include adult patients, or where adult data were not reported separately from pediatric data.
- Studies that only assessed the safety
- Studies that included patients with different groups of PH or where CTEPH patient data could not be separated from data of other PH types.
- Studies that assessed the effect of PH‐targeted therapies together with drugs targeting the NO–sGC–cGMP pathway.
- Case reports with fewer than 10 patients
- Systematic reviews, review articles, conference abstracts without full text, non‐full‐text publications, non‐English articles, opinion papers, and book chapters

**Figure 1:**
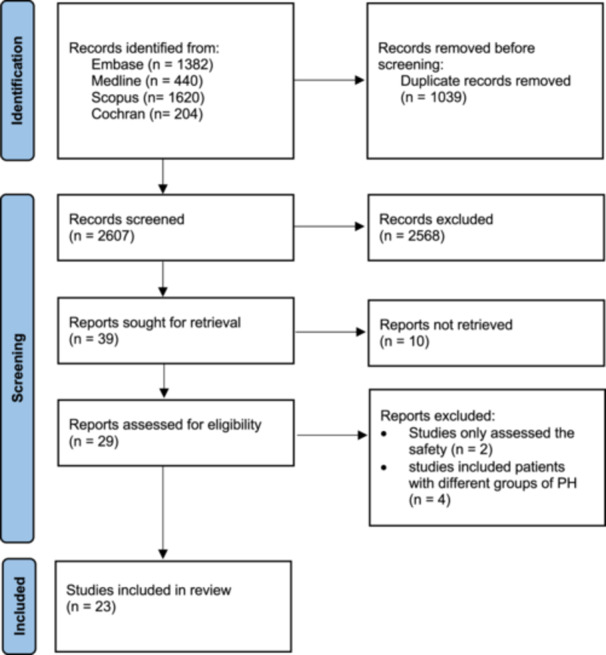
Flowchart illustrating a study selection process based on the Preferred Reporting Items for Systematic Review and Meta‐Analysis Guidelines, including the numbers of studies meeting eligibility criteria and the numbers excluded.

Electronic databases, including Embase, Medline, Cochrane Library, and Scopus were systematically searched from inception to 1 December 2025 to identify studies evaluating drugs targeting the NO–sGC–cGMP pathway (see Supporting Information S1: Table S1 in the Appendix). Studies reporting the effects of these therapies on clinical outcomes in patients with CTEPH were eligible for inclusion in this systematic review. Flowchart illustrating a study selection process is presented in Figure 1.

### 2.2 Study Selection

All retrieved studies were first imported into EndNote and then transferred to the Rayyan platform (https://www.rayyan.ai/) to facilitate blinded screening. Abdullah A. Alqarni and Rawan H. Aljedani independently assessed the titles and abstracts of the identified studies based on the predefined inclusion criteria. When the title and abstract did not provide sufficient information, the full text was reviewed to determine eligibility. Differences between the reviewers were resolved through discussion, and any unresolved disagreements were adjudicated by a third reviewer (Abdulelah M. Aldhahir).

### 2.3 Outcomes

The primary outcomes were changes in exercise capacity primarily measured using the 6‐min walk distance (6MWD), changes in pulmonary hemodynamics, mainly including mean pulmonary arterial pressure (mPAP), pulmonary vascular resistance (PVR), and cardiac output (CO), and changes in WHO‐FC as well as NT‐proBNP levels. The secondary outcomes were quality of life and right ventricular function.

### 2.4 Data Extraction

A standardized data extraction sheet was used to collect data from the eligible full‐text articles. The sheet was prepared and approved by all reviewers before commencing data extraction. Extracted variables included study characteristics (authors, year, country, and study design), participant characteristics (sample size and proportion of males), intervention and comparison groups, reported outcomes, and key findings.

The data extraction was conducted by Abdullah A. Alqarni and Rawan H. Aljedani independently for all included articles, and then the same two reviewers met several times to resolve any disagreements or differences in the extracted data. The completed data extraction sheet was independently verified by two reviewers (Abdulelah M. Aldhahir and Ahmed H. Alasimi). Any discrepancies were resolved through discussion when necessary. The final data extraction sheet was subsequently reviewed by all authors to ensure consistency and accuracy.

### 2.5 Qualitative Assessment of Study Methodology

Two authors (Abdullah A. Alqarni and Rayan A. Siraj) independently completed the assessment of study quality using appropriate tools according to study design. Randomized controlled trials were evaluated using the Cochrane Risk of Bias 2 (RoB 2) tool, while retrospective and observational cohort studies were assessed using the Newcastle–Ottawa Scale.

For randomized controlled trials, the revised Cochrane risk‐of‐bias tool was used [17]. The tool consists of six domains. Each domain included several questions that guided the overall risk‐of‐bias judgment for each study. Studies were classified as low risk when all domains were rated as low risk. A judgment of some concerns was assigned when at least one domain raised concerns, but none were rated as high risk. A study was considered at high risk of bias if at least one domain was rated as high risk or if multiple domains raised concerns (see Supporting Information S1: Tables S2 and S3 in the Appendix).

For retrospective and observational cohort studies, Newcastle–Ottawa Scale which consists of three domains. Each study was awarded a maximum of nine stars based on these domains. Studies scoring 7–9 stars were considered to have low risk of bias (high quality), scores of 4–6 indicated moderate quality, and scores of 0–3 reflected a high risk of bias (see Supporting Information S1: Tables S4 and S5 in the Appendix).

## 3 Results

### 3.1 Description of the Included Studies

Table 1 presents the summary characteristics of the studies included in this systematic review. A total of 23 studies involving 2815 patients were included in this systematic review to assess the therapeutic efficacy of drugs targeting the NO‐sGC‐cGMP pathway in patients with CTEPH [12, 13, 18, 19, 20, 21, 22, 23, 24, 25, 26, 27, 28, 29, 30, 31, 32, 33, 34, 35, 36, 37, 38]. Out of the 23 included studies, most evaluated riociguat, including several randomized controlled trials and large post hoc analyses, while only five studies investigated sildenafil, primarily comprising small‐scale randomized, mechanistic, and observational studies. The final cohort consisted of various study designs, including randomized controlled trials (RCTs), prospective observational or mechanistic studies, retrospective analyses, and post hoc analyses of major clinical trials such as PATENT and CHEST. The principal pharmacological agents investigated were riociguat and sildenafil. These agents were compared with placebo, baseline measurements, or alternative interventions such as BPA. The most frequently evaluated clinical outcomes included exercise capacity, pulmonary hemodynamics, WHO‐FC, NT‐proBNP levels, quality of life, and right ventricular function.

**Table 1:** Summary of studies on drugs targeting the NO‐sGC‐cGMP pathway in patients with CTEPH.

Authors, year, and country	Study design	*n* (male, %)	Intervention vs. comparison	Outcome(s)	Key findings
Thenappan, Al‐Naamani et al. 2020, USA [18]	Post hoc analysis of the PATENT and CHEST studies	697 (26%)	Riociguat vs. placebo	Hemodynamics (PAC and PVR)	Riociguat improved hemodynamics (PAC and PVR)
Wiedenroth, Christoph B. et al. 2018, Germany [19]	Prospective study	36 (61%)	Riociguat vs. baseline	Hemodynamics (mPAP, PVR) NT‐proBNP, and exercise capacity (WHO FC, 6MWD)	Riociguat improved hemodynamics (mPAP, PVR), NT‐proBNP, and exercise capacity (WHO FC, 6MWD)
Zhang, Zhaohua et al. 2024, China [20]	Retrospective study	76 (67%)	Riociguat vs. baseline	Hemodynamics (PVR)	Riociguat improved hemodynamics (PVR)
Ghofrani, H. A et al. 2010, Germany [21]	Prospective, multicentre, open‐label, uncontrolled phase II study	41 (66%)	Riociguat vs. baseline	Exercise capacity, hemodynamics	Riociguat improved exercise capacity and hemodynamics (mPAP, PVR, CI, but not PAWP)
Ahmadi, Ali et al. 2018, Canada [22]	Prospective study	6 (100%)	Riociguat vs. baseline	Right ventricular function (RVSVI, RVEF), myocardial fibrosis, and right ventricular metabolism and perfusion	Riociguat improved Right ventricular stroke volume index; no significant change in myocardial fibrosis, perfusion, or metabolism
Darocha et al., 2018, Poland [23]	Retrospective cohort study	28 (36%)	Sequential therapy: sildenafil followed by riociguat vs. sildenafil alone	Hemodynamics (PVR, mPAP, CO) and WHO FC	Switching from sildenafil to riociguat reduced PVR and mPAP and improved CO and WHO‐FC vs. sildenafil alone.
Ghofrani et al., 2013, Multinational (CHEST‐1) [12]	Phase 3, multicenter, randomized, double‐blind, placebo‐controlled trial	261 (34%)	Riociguat vs. placebo	Exercise capacity (6MWD), hemodynamics (PVR, mPAP, CO), NT‐proBNP, WHO FC	Riociguat improved exercise capacity (6MWD), hemodynamics (PVR, mPAP, CO), NT‐proBNP, and WHO‐FC
Jaïs et al., 2022, France (RACE) [24]	Multicentre, phase 3, open‐label, randomized controlled trial	105 (35%)	Riociguat vs. balloon pulmonary angioplasty (BPA)	Hemodynamics (PVR, mPAP), exercise capacity (6MWD), safety	Riociguat improved hemodynamics (PVR, mPAP) and exercise capacity (6MWD)
Rossi et al., 2008, Italy [25]	Prospective observational study	9 (22%)	Sildenafil vs. baseline	Hemodynamics (mPAP, PVR, CI), exercise capacity (6MWD), endothelial function (endothelin‐1)	Sildenafil improved (mPAP, PVR, CI), exercise capacity (6MWD), and endothelial function (endothelin‐1)
Simonneau et al., 2016, Multinational (CHEST‐2) [13]	Open‐label, long‐term extension study	237 (35%)	Riociguat vs. baseline (long‐term follow‐up)	Exercise capacity (6MWD), NT‐proBNP, WHO‐FC, survival	Long‐term riociguat maintained improvements in exercise capacity (6MWD), NT‐proBNP, and WHO‐FC; 2‐year survival was 93%.
Suntharalingam et al., 2008, UK [26]	Double‐blind, placebo‐controlled pilot RCT with open‐label extension	19 (22%)	Sildenafil vs. placebo (12 weeks), followed by open‐label sildenafil	Exercise capacity (6MWD), WHO‐FC, hemodynamics (mPAP, PVR, CI), NT‐proBNP, quality of life (CAMPHOR)	Sildenafil improved exercise capacity (6MWD), WHO‐FC, hemodynamics (mPAP, PVR, CI), NT‐proBNP, and quality of life
Claessen et al., 2015, Belgium [27]	Prospective mechanistic study with exercise cardiac MRI and invasive hemodynamics	21 (71%)	Sildenafil vs. baseline	Hemodynamic measurements during exercise	Sildenafil improved hemodynamic measurements during exercise
Kawakami et al., 2022, Japan [28]	Open‐label, multicentre, randomized controlled trial	61 (16%)	Balloon pulmonary angioplasty (BPA) vs. riociguat	Pulmonary hemodynamics (mPAP, PVR), exercise capacity (6MWD), WHO‐FC, safety	BPA reduced mPAP and PVR more than riociguat; 6MWD improvements were similar; BPA had more procedural complications and riociguat fewer serious adverse events.
Tatsuo Aoki et al. 2020, Japan [29]	Single‐center, prospective, randomized, open‐label trial	21 (9.52%)	Riociguat vs. control groups	Change in exercise capacity (6MWD) and hemodynamic parameters	Riociguat improved exercise capacity and hemodynamics (mPAP, PVR, and CO) vs. control.
Pavel Jansa et al. 2020, Czech Republic [30]	Retrospective study	51 (45.1%)	Riociguat vs. baseline (single‐arm)	Change in exercise capacity, quality of life questionnaire (EQ. 5D‐5L), and self‐assessment of health status	Riociguat improved exercise capacity, quality of life questionnaire, and self‐reported health status
Nick H. Kim et al. 2016, USA [3]	Post hoc subgroup analysis of the of the CHEST‐1	118 (32%)	Riociguat following PEA vs. control groups	Change in hemodynamic parameters following PEA	Riociguat improved hemodynamic parameters (reduced PVR and mPAP)
F. Reichenberger et al., 2007, Germany [31]	Open‐label uncontrolled clinical trial	104 (43%)	Sildenafil vs. baseline (single‐arm)	Changes in exercise capacity, hemodynamics, and WHO‐FC	Sildenafil improved hemodynamics (PVR and CI), exercise capacity (6MWD), and WHO‐FC from baseline.
Michaela Barnikel et al., 2022, Switzerland [32]	Single‐center, retrospective study	47 (38%)	Riociguat vs. baseline (single‐arm)	Changes in exercise capacity, hemodynamics, and NT pro‐BNP	Riociguat improved exercise capacity, hemodynamics (mPAP, PVR, and CI), and NT pro‐BNP
Mark R. Toshner et al., 2010, UK [33]	Randomized, controlled trial (RCT)	14 (21%)	Sildenafil vs. baseline (single‐arm)	Changes in exercise capacity and NT pro‐BNP	Sildenafil improved exercise capacity and NT pro‐BNP
Cheng‐Hsuan Tsai, et al., 2020, Taiwan [34]	Retrospective observational study	11 (27%)	Riociguat vs. baseline (single‐arm)	Changes in hemodynamics, NT pro‐BNP, WHO‐FC, and exercise capacity (6MWD)	Riociguat improved hemodynamics (mPAP and PVR), NT pro‐BNP, WHO‐FC, but not exercise capacity (6MWD)
Raymond L. Benza et al., 2018 USA [35]	Post hoc analysis of the CHEST‐1 and CHEST‐2 study	237 (17%)	Riociguat vs. placebo groups	Changes in REVEAL risk score	Riociguat improved REVEAL risk score
M.C.J. van Thor et al., 2019 Netherlands [36]	Retrospective study	36 (50%)	Riociguat groups	Changes in exercise capacity, NT‐proBNP, and WHO‐FC	Riociguat improved exercise capacity, NT‐proBNP, and WHO‐FC during follow‐up.
Raymond L. Benza et al., 2021 USA [37]	Post hoc analysis of the PATENT‐1 and CHEST‐1	PATENT‐1 (*n* = 341) and CHEST‐1 (*n* = 238)	Riociguat vs. placebo groups	Changes in right ventricular function	Riociguat improved right ventricular function

Abbreviations: 6MWD, 6‐minute walk distance; BPA, balloon pulmonary angioplasty; CTEPH, chronic thromboembolic pulmonary hypertension; CI, cardiac index; CO, cardiac output; mPAP, mean pulmonary arterial pressure; NT‐proBNP, N‐terminal pro‐brain natriuretic peptide; PAC, pulmonary arterial compliance; PVR, pulmonary vascular resistance; RAP, right atrial pressure; RRS, REVEAL risk score; RV, right ventricle; RVEF, right ventricular ejection fraction; SVI, stroke volume index; WHO‐FC, World Health Organization functional class.

### 3.2 Exercise Capacity

Fifteen studies assessed the impact of NO–sGC–cGMP pathway‐targeted therapies on exercise capacity, primarily measured using 6MWD [12, 13, 19, 21, 24, 25, 26, 28, 29, 30, 31, 32, 33, 34, 36]. Of these, only one retrospective observational study by Tsai et al. reported no significant improvement in 6MWD despite favorable hemodynamic changes with riociguat [34]. In contrast, 14 studies demonstrated significant enhancements in exercise capacity. The multicenter CHEST‐1 trial showed that riociguat significantly improved 6MWD compared with placebo [12]. Furthermore, long‐term follow‐up in the CHEST‐2 study showed that these improvements were maintained over time [13]. Similarly, sildenafil demonstrated efficacy in improving exercise performance. Suntharalingam et al. reported significant improvements in 6MWD in a double‐blind RCT [26], while Reichenberger et al. observed significant baseline‐to‐follow‐up improvements in an open‐label uncontrolled trial study [31]. Overall, 14 of 15 studies reported significant improvements in exercise capacity following treatment with agents targeting the NO–sGC–cGMP pathway in patients with CTEPH.

### 3.3 Pulmonary Hemodynamics

The hemodynamic effects of sGC stimulators and PDE5 inhibitors were extensively examined across 16 studies. Key parameters assessed included mPAP, PVR, and CO [12, 18, 19, 20, 21, 23, 24, 25, 26, 27, 28, 29, 31, 32, 34, 38]. All included studies demonstrated significant improvements in hemodynamic profiles following treatment with riociguat or sildenafil. A post hoc analysis of the PATENT and CHEST trials by Thenappan et al. showed that riociguat significantly improved pulmonary arterial compliance (PAC) and reduced PVR [18]. Additionally, sequential therapy involving a switch from sildenafil to riociguat resulted in additional reductions in mPAP and PVR, along with increased CO [23]. In comparative studies, BPA demonstrated greater reductions in mPAP and PVR compared with riociguat; however, riociguat still provided significant hemodynamic benefits [24, 28]. Sildenafil consistently yielded significant improvements across hemodynamic parameters [25, 26, 27, 31]. Collectively, the evidence indicates that pharmacological modulation of the NO–sGC–cGMP pathway leads to consistent and clinically meaningful improvements in pulmonary hemodynamics in patients with CTEPH.

### 3.4 Biomarkers and Functional Class

Twelve studies evaluated changes in NT‐proBNP levels and WHO‐FC as markers of therapeutic response [12, 13, 19, 23, 26, 28, 31, 32, 33, 34, 35, 36]. Riociguat therapy was associated with significant reductions in NT‐proBNP levels and improvements in WHO‐FC in both the short‐term CHEST‐1 trial [12] and its long‐term extension, CHEST‐2 [13]. Comparable findings were observed with sildenafil, as Reichenberger et al. reported sustained improvements in WHO‐FC with long‐term treatment [31]. Taken together, agents targeting the NO–sGC–cGMP pathway effectively improved functional status and reduced biomarker indicators of disease severity in patients with CTEPH.

### 3.5 Quality of Life

Patient‐reported outcomes, particularly health‐related quality of life, were evaluated in two of the included studies [26, 30]. In a retrospective registry analysis, riociguat was significantly associated with improved quality of life scores as measured by the EuroQol 5 Dimensions 5 Levels questionnaire (EQ‐5D‐5L), alongside enhanced self‐reported health status [30]. Moreover, Suntharalingam et al. demonstrated that sildenafil significantly improved quality of life, as assessed by the Cambridge Pulmonary Hypertension Outcome Review scale during the open‐label extension phase of their pilot study [26]. Overall, the evidence consistently suggests that drugs targeting the NO‐sGC‐cGMP pathway positively influence patient‐reported well‐being in CTEPH.

### 3.6 Right Ventricular Function and Risk Assessment

Three of the included studies examined the effects of NO–sGC–cGMP pathway‐targeted therapies on right ventricular function and global risk scores [22, 35, 37]. A post hoc analysis of the PATENT‐1 and CHEST‐1 trials demonstrated that riociguat significantly improved RV function [37]. In a prospective mechanistic study, Ahmadi et al. reported that riociguat significantly increased the right ventricular stroke volume index, although no significant changes were observed in myocardial fibrosis, perfusion, or metabolic parameters [22]. Together, these findings suggest that therapies targeting the NO–sGC–cGMP pathway confer measurable benefits in cardiac function among patients with CTEPH.

A post hoc analysis by Benza et al. from CHEST‐1/CHEST‐2, evaluated REVEAL risk score in CTEPH and found that riociguat improved REVEAL risk score compared with placebo [35]. However, as this evidence comes from a single post hoc study and REVEAL was originally developed for PAH rather than CTEPH, its applicability in CTEPH should be interpreted cautiously.

## 4 Discussion

The findings of this systematic review demonstrated that therapeutic modulation of the NO–sGC–cGMP signaling pathway provides consistent clinical benefit for patients with CTEPH pathobiology. Across 23 studies involving 2815 patients, agents targeting this pathway, most notably riociguat and sildenafil, were associated with improvements in exercise capacity, pulmonary hemodynamics, biomarkers of cardiac strain, functional status, right ventricular performance, and, where evaluated, health‐related quality of life [12, 13, 21]. These findings underscore the central role of NO‐sGC‐cGMP pathway in CTEPH pathobiology and support the use of pathway‐targeted therapies in patients with persistent or recurrent PH following PEA [4, 5].

Improvement in 6MWD, reported in 14 of the 15 included studies, highlights the ability of NO‐sGC‐cGMP‐enhancing therapies to improve functional capacity. The 6MWT remains the most widely used clinical measure of submaximal exercise performance in CTEPH, and consistent gains observed across studies suggested enhancement of physiologic reserve. Across the included studies, both riociguat and sildenafil were associated with improvement in 6MWD, reflecting enhanced functional capacity and physiologic reserve [23]. Findings from the CHEST‐1 randomized controlled trial demonstrated that riociguat significantly increased 6MWD compared with placebo, with mean improvements of 39–46 m over 16 weeks [39]. Growing evidence indicates that improvements in 6MWD reflect enhancement in right ventricular‐pulmonary arterial coupling, ventilatory efficiency, and peripheral oxygen extraction, indicating that the functional gains achieved with NO–sGC–cGMP‐targeted therapy extend beyond symptomatic relief and correspond to meaningful cardiopulmonary remodeling [40].

Differences in the magnitude of benefit observed between agents may relate to their pharmacologic mechanisms. Riociguat directly stimulates sGC, thereby enhancing cGMP production independently of endogenous NO, whereas sildenafil inhibits PDE5‐mediated degradation of cGMP [41]. These mechanistic distinctions may contribute to variability in hemodynamic response, onset of action, and antiproliferative activity across patient subgroups. Beyond monotherapy, emerging evidence indicates that sequential combination therapy with long‐term riociguat followed by BPA further improves pulmonary hemodynamics, right ventricular function, and exercise capacity, highlighting potential additive benefits when integrating targeted medical therapy with interventional approaches in inoperable CTEPH [42]. Furthermore, a recent post hoc analysis of the RACE trial further showed that both riociguat and BPA improved right ventricular afterload in inoperable CTEPH [43]; however, BPA produced a greater reduction in afterload, and improvement in right ventricular function was observed only with BPA. Further research is required to determine optimal sequencing strategies, patient selection criteria, and long‐term outcomes associated with combined modalities.

Although only two studies in this review formally assessed patient‐reported outcomes [26, 30], additional evidence supports the impact of NO‐sGC‐cGMP pathway modulation on quality of life. A recent meta‐analysis in CTEPH demonstrated a significant improvement in EQ‐5D‐5L and living with PH scores with riociguat compared with placebo, aligning symptomatic benefit with functional and hemodynamic gains [44]. These findings are consistent with a meta‐analysis of randomized controlled trials in PH, which reported that riociguat improved both 6MWD and EQ‐5D‐5L scores [45], indicating that functional enhancement is closely linked to improved patient‐perceived well‐being.

Although improvements in patient‐related outcomes and functional capacity highlight the clinical benefits of NO–sGC–cGMP‐targeted therapy, these effects may partly reflect underlying cardiac adaptations. Preclinical evidence demonstrates that modulation of this pathway exerts direct beneficial effects on the right ventricular function independent of pulmonary afterload. In a pressure‐overload model of right ventricular hypertrophy, both riociguat and sildenafil prevented deterioration of right ventricular function—evidenced by reduced RV dilation, improved ejection fraction, and preserved stroke volume—compared with placebo [46]. Riociguat further attenuated right ventricular fibrosis, suggesting anti‐remodeling and cardioprotective effects under sustained pressure overload. These findings support the biological plausibility of improved right ventricular performance observed clinically and emphasize the importance of right ventricular adaptation as a key determinant of outcomes in CTEPH.

Importantly, right ventricular adaptation represents a major determinant of prognosis in CTEPH, and conductance‐catheter pressure–volume analysis has demonstrated that thromboembolic obstruction leads to marked impairments in right ventricular‐pulmonary arterial coupling, diminished contractile reserve, and systolic dysfunction. Experimental evidence from the sGC stimulator riociguat ameliorates PH induced by hypoxia and su5416 in rats [47]. This provides mechanistic therapies that improve right ventricular structure and function, which may offer prognostic benefits extending beyond hemodynamic or symptomatic improvement [48]. The observed direct myocardial effects of NO–sGC–cGMP modulation provide mechanistic plausibility for such long‐term benefit.

Although this review focused primarily on efficacy, safety considerations are clinically important in interpreting NO–sGC–cGMP pathway‐targeted therapies in CTEPH. Riociguat is generally well tolerated in CTEPH, with pooled evidence suggesting no significant excess of hypotension versus placebo, although common adverse events such as dyspepsia and peripheral edema have been reported [44]. Sildenafil is also usually well tolerated and generally causes only a small reduction in systemic blood pressure [49]. However, cautious use of sildenafil is warranted in patients at risk for systemic hypotension or bleeding.

### 4.1 Strengths and Limitations

This review represents one of the most comprehensive evaluations of NO–sGC–cGMP–targeted therapies in CTEPH, integrating randomized controlled trials, prospective cohorts, and real‐world observational studies assessing hemodynamic, functional, biomarker, right‐ventricular, and quality‐of‐life outcomes. However, several limitations should be acknowledged. The study heterogeneity remains a key limitation. Variability in baseline disease severity, prior PEA or BPA status, treatment duration, and outcome reporting complicates direct comparisons across studies. Furthermore, long‐term data remain limited, and standardized assessment of right ventricular function and patient‐reported outcomes is lacking. In addition, that safety outcomes were not uniformly reported across the included studies, particularly for bleeding events, which limited a more comprehensive comparison of the safety profile of NO–sGC–cGMP pathway‐targeted therapies in CTEPH.

### 4.2 Future Direction

Targeting the NO–sGC–cGMP pathway is an established therapeutic strategy in PH, with riociguat approved for CTEPH and supported by pivotal trials such as CHEST‐1 and its long‐term extension CHEST‐2, which demonstrated sustained improvements in exercise capacity and functional class [50, 51]. The findings of this systematic review, together with real‐world and observational data, further suggest that agents such as riociguat and sildenafil can improve pulmonary hemodynamics, functional status, and clinical outcomes, including reductions in PVR and improvements in CO [23, 52]. However, with the expanding role of interventional strategies such as BPA and emerging evidence supporting sequential or combination therapy, additional studies are required to determine optimal treatment sequencing and patient selection [53]. Future investigations should focus on standardized outcome measures, long‐term efficacy, and the development of novel therapeutic approaches, including combination regimens and alternative delivery methods, to further optimize clinical outcomes in patients with CTEPH.

## 5 Conclusion

Drugs targeting the NO–sGC–cGMP pathway, particularly riociguat, show consistent improvements in exercise capacity, hemodynamics, and functional outcomes in CTEPH. The evidence for riociguat is robust and guideline‐supported, especially in inoperable or persistent/recurrent disease after PEA. In contrast, evidence for sildenafil is limited and should be interpreted with caution due to the small number of studies. Further high‐quality trials are needed to confirm its efficacy. Pharmacological therapy remains an important component of management, alongside interventional approaches such as BPA.

## Author Contributions

Abdullah A. Alqarni, Hassan Alwafi, Rawan H. Aljedani, Ahmed H. Alasimi, and Abdulelah M. Aldhahir contributed to the conception and design of the review. Abdullah A. Alqarni, Rawan H. Aljedani, Abdulelah M. Aldhahir, Rayan A. Siraj, and Ahmed H. Alasimi contributed to data screening and extraction. Abdullah A. Alqarni, Abdulkareem A. AlGarni, Rawan H. Aljedani, Abdulelah M. Aldhahir, Ahmed H. Alasimi, Jaber S. Alqahtani, Saeed Mardy Alghamdi, Rayan A. Siraj, Aminah Mengash, and Hassan Alwafi interpreted data and wrote sections of the manuscript. All authors contributed to the manuscript's revision and read and approved the submitted version.

## Ethics Statement

The authors have nothing to report.

## Consent

The authors have nothing to report.

## Conflicts of Interest

The authors declare no conflicts of interest.

## Supporting information

Supporting File

## Data Availability

All data generated and analyzed during this study are available from the corresponding author upon reasonable request.
