# Supplementary material for: The Therapeutic Efficacy of Drugs Targeting the NO‐sGC‐cGMP Pathway in Treatment of Patients With Chronic Thromboembolic Pulmonary Hypertension: A Systematic Review
Source: Pulm Circ. 2026 Jul 15;16(3):e70337. doi: 10.1002/pul2.70337 (PMC13404768; doi:10.1002/pul2.70337)
Supplement: Supplementary file 1 — Supporting File [file PUL2-16-e70337-s001.pdf]

Supplementary Material

Title: The therapeutic efficacy of drugs targeting the NO-sGC-cGMP pathway in treatment of patients with chronic thromboembolic pulmonary hypertension: a systematic review

Research question: Does the use of drugs targeting the NO-sGC-cGMP pathway improve clinical outcomes in patients with chronic thromboembolic pulmonary hypertension?

Search words

Intervention: inhaled nitric oxide, nitric oxide, NO, inhaled NO, soluble guanylate cyclase stimulator, sGC stimulator, Riociguat, inhaled soluble guanylate cyclase stimulator, inhaled sGC stimulator, MK-5475, sildenafil, revatio, tadalafil, adcirca, PDE5 inhibitor, phosphodiesterase type 5 inhibitor

Participants: pulmonary hypertension, PAH, group 4 pulmonary hypertension, chronic thromboembolic pulmonary hypertension, CTEPH, group 4 PH, pulmonary hypertension due to chronic thromboembolism

Appendices

Search strategy for the systematic review

Table S1: Search strategy in Embase, Medline, Cochrane and Scopus

Medline and Embase (through Ovid)	
1	exp Hypertension/ 326753
2	(hypertension or (blood adj2 pressure*)).kf,fx,tw. 732210
3	exp Hypertension, Pulmonary/ 43807
4	(Pulmonary adj2 hyperten*).kf,fx,tw.59523
5	exp Thromboembolism/ 66378
6	((group 4 or group four) adj3 (pulmonary adj2 hypertension)).kf,fx,tw. 48
7	(group 4 adj2 PH).kf,fx,tw. 65
8	((chronic adj4 thrombo* adj3 pulmonary adj2 hypertension) or CTEPH).kf,fx,tw. 3490
9	1 or 2 or 5 860582
10	3 or 4 67599
11	6 or 7 or 8 3545
12	9 and 10 and 11 3506
13	exp nitric oxide/ 98114
14	"nitric oxide* ".kf,fx,tw. 171613
15	(genosyl or inomax or noxivent).kf,fx,tw. 12
16	nitrogen monoxide.kf,fx,tw. 659
17	(NO adj3 inhal*).kf,fx,tw. 2459
18	13 or 14 or 15 or 16 or 17 190783
19	(guanylate cyclase or sGC or MK-5475 or riociguat).kf,fx,tw. 13520
20	exp antihypertensive agent/ 273853
21	exp sildenafil/ 6012
22	exp tadalafil/ 1784
23	(sildenafil or tadalafil or revatio or adcirca).kf,fx,tw. 9548
24	(Phosphodiesterase-5 or PDE-5 or Phosphodiesterase-V or PDE-V).kf,fx,tw. 4189
25	((Phosphodiesterase or PDE) adj3 five).kf,fx,tw. 56
26	exp Guanylate Cyclase/ 9658
27	19 or 26 18581
28	21 or 22 or 23 10703
29	exp Phosphodiesterase 5 Inhibitors/ 9481
30	24 or 25 or 29 11496
31	27 or 28 or 30 32027
32	20 or 31 303202
33	exp Cyclic GMP/ 22973

34	(cyclic guanosine monophosphate or cGMP).kf,fx,tw. 26238
35	33 or 34 35827
36	32 or 35 327874
37	18 or 36 494024
38	12 and 37 441
39	exp Hypertension/ 326753
40	(hypertension or (blood adj2 pressure*)).kf,fx,tw. 732210
41	exp Hypertension, Pulmonary/ 43807
42	(Pulmonary adj2 hyperten*).kf,fx,tw.59523
43	exp Thromboembolism/ 66378
44	((group 4 or group four) adj3 (pulmonary adj2 hypertension)).kf,fx,tw. 48
45	(group 4 adj2 PH).kf,fx,tw. 65
46	((chronic adj4 thrombo* adj3 pulmonary adj2 hypertension) or CTEPH).kf,fx,tw. 3490
47	39 or 40 or 43 860582
48	41 or 42 67599
49	44 or 45 or 46 3545
50	47 and 48 and 49 3506
51	exp nitric oxide/ 98114
52	"nitric oxide* ".kf,fx,tw. 171613
53	(genosyl or inomax or noxivent).kf,fx,tw. 12
54	nitrogen monoxide.kf,fx,tw. 659
55	(NO adj3 inhal*).kf,fx,tw. 2459
56	51 or 52 or 53 or 54 or 55 190783
57	(guanylate cyclase or sGC or MK-5475 or riociguat).kf,fx,tw. 13520
58	exp antihypertensive agent/ 273853
59	exp sildenafil/ 6012
60	exp tadalafil/ 1784
61	(sildenafil or tadalafil or revatio or adcirca).kf,fx,tw. 9548
62	(Phosphodiesterase-5 or PDE-5 or Phosphodiesterase-V or PDE-V).kf,fx,tw. 4189
63	((Phosphodiesterase or PDE) adj3 five).kf,fx,tw. 56
64	exp Guanylate Cyclase/ 9658
65	57 or 64 18581
66	59 or 60 or 61 10703
67	exp Phosphodiesterase 5 Inhibitors/ 9481
68	62 or 63 or 67 11496
69	65 or 66 or 68 32027
70	58 or 69 303202
71	exp Cyclic GMP/ 22973
72	(cyclic guanosine monophosphate or cGMP).kf,fx,tw. 26238
73	71 or 72 35827
74	70 or 73 327874
75	56 or 74 494024
76	50 and 75 441

Scopus
(ALL(hypertension OR (*blood AND pressure*)) AND TITLE-ABS-KEY((*pulmonary AND hyperten*) OR "pulmonary hypertension" OR (thromboe* AND (*pulmonary AND hyperten*) OR (group 4 AND (*pulmonary AND hyperten*) OR group 4 pulmonary AND hypertension OR group 4 ph))) AND TITLE-ABS-KEY((chronic AND thrombo* AND pulmonary AND hypertension) OR cteph OR (ulmonary AND hypertension AND chronic AND thrombo*)) AND TITLE-ABS-KEY((nitric AND oxide*) OR genosyl OR inomax OR noxivent OR (nitrogen AND monoxide) OR (inhal* AND nitric AND oxid*)) OR TITLE-ABS-KEY((guanylate AND cyclase AND activator) OR (guanylate AND cyclase) OR sgc OR mk-5475 OR riociguat OR sildenafil OR tadalafil OR revatio OR adcirca) OR TITLE-ABS-KEY((phosphodiesterase AND v AND inhibitor) OR (phosphodiesterase-5 OR pde-5 OR phosphodiesterase-v OR pde-v) OR (phosphodiesterase OR pde) OR ((phosphodiesterase OR pde) five) OR (cyclic AND guanosine AND monophosphate) OR cgmp) OR TITLE-ABS-KEY((antihypertensive AND agent))) AND (LIMIT-TO (LANGUAGE,"English"))
**Cochrane**<br>Search Name: Abdullah SR3

ID	Search Hits
#1	Hypertension 80685
#2	(hypertension or (blood adj2 pressure*)) 81080
#3	MeSH descriptor: [Hypertension, Pulmonary] explode all trees 1703
#4	MeSH descriptor: [Pulmonary Embolism] explode all trees 1536
#5	group 4 pulmonary hypertension 3024
#6	((group 4 or group four) NEXT (pulmonary hypertension)) 5029
#7	chronic thrombo* pulmonary hypertension 732
#8	#1 OR #2 OR #3 Or #4 Or #5 OR #6 84302
#9	#8 AND #7 732
#10	MeSH descriptor: [Nitric Oxide] explode all trees 2767
#11	(nitric oxide):ti,ab,kw 9506
#12	(genosyl or inomax or noxivent):ti,ab,kw 14
#13	MeSH descriptor: [Nitric Oxide] explode all trees 2767
#14	(NO inhal*) 18436
#15	#10 OR #11 OR #12 OR #13 OR #14 26503
#16	(guanylate cyclase or sGC or MK-5475 or riociguat):ti,ab,kw 655
#17	MeSH descriptor: [Sildenafil Citrate] explode all trees 1188
#18	MeSH descriptor: [Tadalafil] explode all trees 615
#19	(sildenafil or tadalafil or revatio or adcirca):ti,ab,kw 3460
#20	MeSH descriptor: [Guanylate Cyclase] explode all trees 78
#21	#16 or #20 669
#22	#17 OR #18 OR #19 3460
#23	(Phosphodiesterase-5 or PDE-5 or Phosphodiesterase-V or PDE-V):ti,ab,kw 1167

#24	((Phosphodiesterase or PDE) five)	382
#25	MeSH descriptor: [Phosphodiesterase 5 Inhibitors] explode all trees	544
#26	#23 OR #24 OR #25	1464
#27	MeSH descriptor: [Cyclic GMP] explode all trees	305
#28	(cyclic guanosine monophosphate or cGMP):ti,ab,kw	820
#29	#27 OR #28	914
#30	MeSH descriptor: [Antihypertensive Agents] explode all trees	10908
#31	#15 OR #21 OR #22 OR #26 OR #29 OR #30	41876
#32	#9 AND #31	205

Table S2. Summary of the Cochrane Risk-of-Bias Tool (RoB2)

Domain	Ghofrani 2013	Jaïs 2022	Suntharalingam 2008	Kawakami 2022	Aoki 2020	Toshner 2010
**Randomization process**	Low	Low	Low	Low	Some concerns	Some concerns
**Deviations from intended interventions (assignment)**	Low	High	Low	High	High	High
**Deviations from intended interventions (adherence)**	Low	High	Low	High	High	High
**Missing outcome data**	Low	Low	Low	Low	Low	Low
**Measurement of outcome**	Low	Low	Low	Low	Low	Low
**Selection of reported result**	Low	Low	Low	Low	Low	Low
**Overall bias**	**Low**	**High**	**Low**	**High**	**High**	**High**

Table S3. Visual Risk-of-Bias Summary

Study	Overall Risk
**Ghofrani 2013**	🟢 Low
**Jaïs 2022**	🔴 High
**Suntharalingam 2008**	🟢 Low
**Kawakami 2022**	🔴 High
**Aoki 2020**	🔴 High
**Toshner 2010**	🔴 High

Table S4. Newcastle–Ottawa Scale Assessment of Observational Studies

Study	Selection (4)	Comparability (2)	Outcome (3)	Total (9)	Quality
**Thenappan 2020**	4	2	3	**9**	High
**Wiedenroth 2018**	3	1	3	**7**	High
**Zhang 2024**	3	1	2	**6**	Moderate
**Ghofrani 2010**	3	1	2	**6**	Moderate
**Ahmadi 2018**	2	1	2	**5**	Moderate
**Darocha 2018**	3	2	2	**7**	High
**Rossi 2008**	2	1	2	**5**	Moderate
**Simonneau 2016 (CHEST-2)**	4	2	3	**9**	High
**Claessen 2015**	3	1	2	**6**	Moderate
**Jansa 2020**	3	1	2	**6**	Moderate
**Reichenberger 2007**	3	1	2	**6**	Moderate
**Barnikel 2022**	3	1	2	**6**	Moderate
**Tsai 2020**	2	1	2	**5**	Moderate
**van Thor 2019**	3	1	2	**6**	Moderate
**Kim 2016**	4	2	3	**9**	High
**Benza 2018**	4	2	3	**9**	High
**Benza 2021**	4	2	3	**9**	High

Table S5. Visual summary of Newcastle–Ottawa Scale assessment

Study	Overall quality
**Thenappan 2020**	High
**Wiedenroth 2018**	High
**Zhang 2024**	Moderate
**Ghofrani 2010**	Moderate
**Ahmadi 2018**	Moderate
**Darocha 2018**	High
**Rossi 2008**	Moderate
**Simonneau 2016**	High
**Claessen 2015**	Moderate
**Jansa 2020**	Moderate
**Reichenberger 2007**	Moderate
**Barnikel 2022**	Moderate
**Tsai 2020**	Moderate
**van Thor 2019**	Moderate
**Kim 2016**	High
**Benza 2018**	High
**Benza 2021**	High
